# Monitoring and Assessment of Indoor Environmental Conditions after the Implementation of COVID-19-Based Ventilation Strategies in an Educational Building in Southern Spain

**DOI:** 10.3390/s21217223

**Published:** 2021-10-30

**Authors:** Antonio J. Aguilar, María L. de la Hoz-Torres, Mª Dolores Martínez-Aires, Diego P. Ruiz

**Affiliations:** 1 Department of Applied Physics, University of Granada, Av. Severo Ochoa s/n, 18071 Granada, Spain; antojes@ugr.es (A.J.A.); mlhoz@ugr.es (M.L.d.l.H.-T.); 2 Department of Building Construction, University of Granada, Av. Severo Ochoa s/n, 18071 Granada, Spain; aires@ugr.es

**Keywords:** indoor environmental quality, buildings, natural ventilation, COVID-19, IEQ, educational buildings, construction

## Abstract

Since students and teachers spend much of their time in educational buildings, it is critical to provide good levels of indoor environmental quality (IEQ). The current COVID-19 pandemic has shown that maintaining a good indoor air quality level is an effective measure to control the transmission of the SARS-CoV-2 virus. This study used sensors to monitor key IEQ factors and assess several natural ventilation scenarios in a classroom of the University of Granada. Subsequently, the IEQ factors (temperature, relative humidity, CO_2_ concentration, acoustic environment, and air velocity) were evaluated for the selected ventilation scenarios in the occupied classroom, and the field monitoring was carried out in two different assessment periods, winter and summer. The obtained results show that the CO_2_ concentration levels were well below the recommended limits. However, the maintenance of the recommended thermal and acoustic IEQ factors was significantly affected by the natural ventilation strategies (temperature and relative humidity values were very close to the outside values, and the background sound pressure level was over 35 dBA during the entire assessment). The proper measurements and careful selection of the appropriate ventilation scenarios become of utmost importance to ensure that the ventilation rates required by the health authorities are achieved.

## 1. Introduction

Nowadays, the global energy scenario shows that buildings consume more than twice as much energy as they did in 1970 and account for 40% of the energy consumed at present [1,2]. The increase in global energy demand, along with the climate crisis, has become a major public concern. This situation has led to a focus on research and policy efforts to ensure environmental and energy efficiency [3], as well as wellbeing and health conditions [4]. In this context, the building sector faces the challenge of reducing energy consumption and greenhouse gas emissions, thereby minimising the environmental impact of buildings whilst maintaining indoor environmental conditions that are suitable to the health and safety of users [5]. The environmental performance of buildings depends, among other factors, on the interface between the occupants and the physical environment [6].

As part of the building stock, educational buildings represent a noteworthy part of the total energy use [7]. In fact, providing adequate indoor environmental conditions in educational buildings requires extensive energy consumption. If the building is not able to maintain adequate indoor thermal conditions to achieve thermal comfort (TC), this results in an increase in energy demand [8]. As public buildings, this characteristic gives them a major social responsibility. Thus, the energy performance and appropriate levels of indoor environmental quality (IEQ) (such as TC, indoor air quality (IAQ), and acoustic comfort (AC)) in these buildings are of great importance [9]. Buildings not only have to meet the standards required for an indoor environment but it is also essential that they ensure the health and comfort of their occupants [6].

In this regard, it is worth mentioning that IEQ has attracted the attention of researchers as one of the characteristics of sustainable buildings and the environment as the behaviour of the occupants and users of the building are seriously affected by it [6]. Previous research has shown strong evidence that relates inadequate IEQ with adverse health effects, illness, wellbeing, and reduced productivity [10]. Since students and teachers spend much of their time in educational buildings, it is critical to provide good TC, AC, and IAQ levels. Poor indoor TC can create unsatisfactory conditions, and it may have a negative influence on students’ learning and performance [11,12,13]. In this regard, thermal discomfort can influence students’ capacity to learn [14]. Indeed, previous research has shown that increases in classroom temperature significantly decrease the performance levels of students [15,16], resulting in a negative impact on students’ ability to complete tasks and their ability to learn [17]. AC is also essential in learning spaces. A poor acoustic environment in classrooms can affect students’ academic, psychosocial, and psycho-educational achievement. However, it does not only affect students but can also cause voice problems [18] and physical stress for the teacher [19]. In addition, many research studies highlight that poor IAQ may affect the comfort, productivity, and academic achievement of students [20,21,22]. The exposure to air pollutants may cause many diseases, such as irritated eyes or nose, blocked nose, headache and so forth [23], cardiovascular disease [24], and several respiratory diseases [25,26].

Therefore, evidence from previous studies highlights the relevance of the indoor environmental conditions in learning spaces. However, the achievement of a good environment is often not considered a priority in the design of educational buildings [27]. In this endeavour of ensuring adequate IAQ inside classrooms, the renewal of indoor air through ventilation is required. However, as stated by Becker et al. [28], the ventilation of educational buildings in warm climates has a dilemma between the IAQ and TC on one hand and energy efficiency on the other. A sufficiently high ventilation rate (VR) with outdoor air is needed so as to not compromise the IAQ and to remove pollutants emitted from indoor sources [29]. If the VRs are reduced, the IAQ deteriorates but energy is saved simultaneously [30]. Given the relevance of both criteria, previous studies have analysed strategies to improve their energy and environmental efficiencies whilst ensuring health conditions [31].

Nevertheless, the current circumstances arising from the COVID-19 health crisis have meant that ensuring that spaces are safe, and health is a priority over comfort or energy efficiency. The growing concern over the indoor environmental conditions inside buildings, with a particular focus on teaching–learning spaces [32], has led governments to take action in these buildings through the provision of adequate IAQ. The partial and full closures of educational buildings and the limited access to learning spaces (classrooms, laboratories, and related spaces) are factors that have conditioned the learning process. The IEQ conditions, especially with respect to the IAQ, have been particularly important aspects regarding the reopening of these spaces in the context of the COVID-19 pandemic. 

In this context, the Ministry of Health of the Spanish Government stated that the proper ventilation of indoor spaces is a measure to minimise the transmission of SARS-CoV-2 through aerosols [33]. The recommendations and preventive measures include, among others, that the VR should be ~5–6 air change per hour (ACH) in order not to compromise good air quality. This VR value can be achieved by increasing the flow of outside air provided by either natural means (opening windows and doors for the time deemed necessary according to the characteristics of each space) or mechanical means. In those classrooms that do not have a mechanical ventilation system, the Contingency and Action Plan for COVID-19 [34] drawn up by the University of Granada (which is similar to other action plans in Europe) establishes that “even if the weather conditions are adverse, ventilation must be carried out by means of natural ventilation through open windows and doors”, which is one of the necessary measures of ventilation and air conditioning requirements for the reopening of educational centres. In addition, the classroom must be naturally ventilated at least 1 h before and after the teaching activity takes place. In this sense, the correct selection of the window and door opening configuration in teaching spaces is crucial to achieve a balance between optimal ventilation conditions and comfort for the users who occupy the spaces. Nevertheless, previous research has shown that natural ventilation strategies that provide the required VR may have a high impact on other variables, such as indoor acoustic comfort [35]. The developed tests in [35] were conducted in unoccupied classrooms, and the impact on the acoustic quality of the interior space in several classrooms was analysed.

This research is framed in the above context, and, even though many studies have reported on the impact of natural ventilation on building performance, there has been very little research reported on the impact of implementing the recommended VR through natural ventilation strategies to prevent air-borne disease transmission. The purpose of the present study is, therefore, to assess the impact of those measured states by the Spanish government in the IEQ of educational buildings by using environmental sensors to monitor relevant IEQ factors (temperature, relative humidity, CO_2_ concentration, acoustic environment, and air velocity). Unlike previous studies, such as in De la Hoz-Torres et, al, 2021 [35], the classroom is monitored “in use”, and, more specifically, during examination periods. Since poor environmental condition exposure is associated with a reduction of cognitive performance and deterioration of physical and mental health [36], the monitoring in scenarios where students are taking exams can provide valuable information to assess the potential impact on their performance. In this way, the results obtained in this case study can support the decision-making process of building managers in managing the best suitable natural ventilation strategies.

To achieve this goal, this study includes both an experimental part of sensor data collection and a subsequent analysis section. This work aims to measure and assess ventilation rates (VR) in addition to other factors, such as air temperature, relative humidity, air velocity, and CO_2_ concentration, in several different scenarios in a classroom with VR conditions recommended by Spanish public guidelines. The acquired data and their analysis are the contribution of this work, and the proposal will contribute as a basis to defining guidelines regarding ventilation strategies that meet the established safety standards against airborne disease transmission and improve the IAQ in learning spaces. Moreover, and in a second instance, the data can also be used to analyse the level of IEQ and as a basis for future research to propose optimised systems in terms of natural ventilation and user safety and comfort.

## 2. Materials and Methods

As noted in the previous section, this research deals with indoor environmental conditions in teaching–learning spaces. Specifically, the present study aims to investigate how the Spanish government requirements on ventilation strategies will affect indoor environmental conditions, to assess their impact, and establish appropriate actions. In this sense, for the assessment of the impact on the IEQ due to increased ventilation at the rates required by the public health recommendations, experimental tests were carried out in different natural ventilation scenarios. Subsequently, the field measurements were carried out in two assessment periods: winter and summer. 

### 2.1. Description of Case Study Area and Climatic Conditions

For the purposes of the current study, teaching spaces of the Fuentenueva campus of the University of Granada were investigated. This campus is located in the urban area of Granada (37°11′ N, 3°36′ W). This Spanish city, located in the southeast of the peninsula, has a Mediterranean climate with hot and dry summers and cool damp winters. It is also characterised by strong daily and seasonal variations of temperature [37]. The daily temperatures average 34 °C in the hottest month of the year (July) and 13 °C in the coldest (January). However, the temperatures during the night can drop to ~1 °C in the coldest month.

Since on-campus and face-to-face educational provisions were not possible from October to January due to the health crisis caused by COVID-19, extraordinary measures for off-campus teaching and learning were adopted. After the state of health emergency ended, and with the aim of providing safe and healthy spaces for the return to campus, the University of Granada developed the COVID-19 Action Plan [34]. The measures defined in the plan include mandatory masks indoors, physical distancing (1.5 m at least), and capacity limited to 50%. In this sense, in order to implement these measures for the return to face-to-face teaching, it is necessary to use sufficiently large spaces to guarantee these requirements. In addition, the plan states that natural ventilation will be maintained via open windows and doors even if the weather conditions are adverse. Therefore, health criteria take precedence above energy efficiency and conditions of comfort [34].

**Figure 1:**
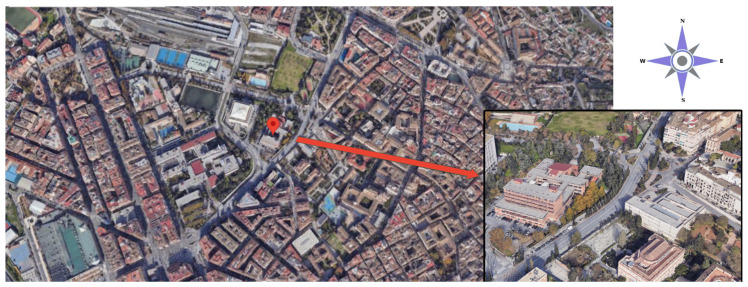
Location of case study.

In this context, a typical classroom was selected from those that met the requirements set out in the COVID-19 Action Plan, i.e., a classroom located in the Advanced Technical School for Building Engineering (Figure 1). This classroom is a representative case study of the classrooms used after returning to the campus due to its typical characteristics in terms of geometry, equipment, and capacity. The tests were all conducted in this classroom.

**Figure 2:**
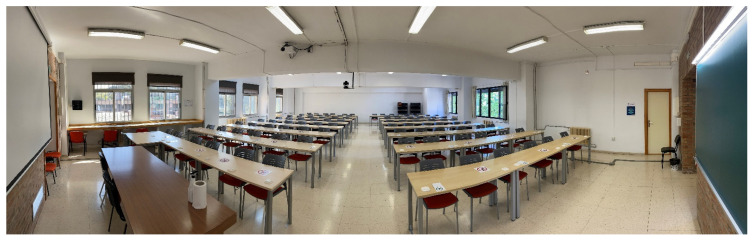
Selected classroom.

The classroom does not have a mechanical ventilation system; therefore, natural ventilation is the only possible mechanism for exchanging the air inside the room (Figure 2). The area and volume of the classroom are 175 m^2^ and 524 m^3^, respectively. It contains five windows in the northeast façade, with dimensions of 1.80 m × 2.00 m. In addition, there are two windows in the southwest façade, with dimensions of 2.50 m × 2.10 m. The access to the classroom is through a main door, with dimensions of 1.50 m × 2.00 m.

Different window and door opening configurations were defined to assess the VR in the classroom. The different opening configurations of northeast-facing windows, southwest-facing windows, and the main door provide three natural cross-ventilation scenarios defined for this research: (1) Ventilation Scenario 1 was constructed with five northeast-facing windows open and two southwest-facing windows open. (2) Ventilation Scenario 2 was constructed with two windows open on both the northeast and southwest façades. (3) Ventilation Scenario 3 was constructed with two northeast-facing windows open and only one southwest-facing window open. In all three ventilation scenarios, the main door was open.

### 2.2. Sensor Location and Data Collection: Natural Ventilation Strategies Setup

**Figure A1:**
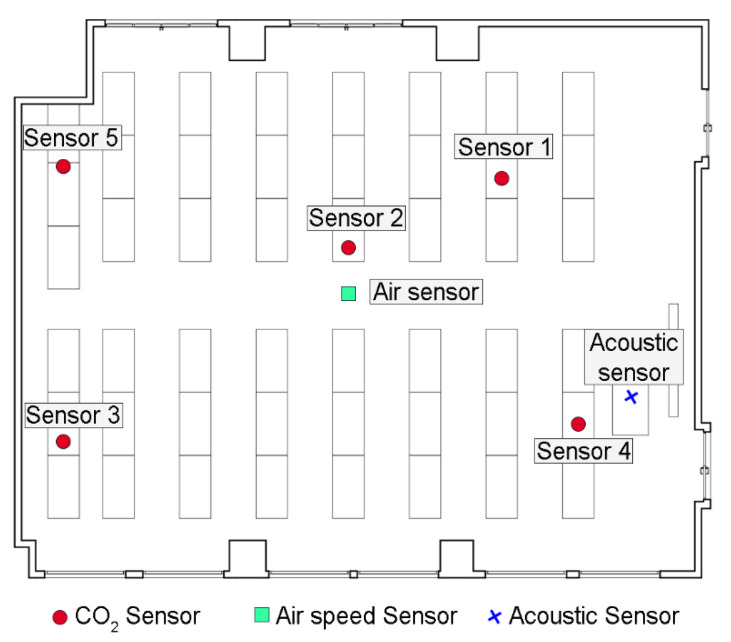
Classroom layout and field measurement configuration.

In order to quantify the natural VR under different configuration of doors and/or windows opening conditions, the tracer gas decay method was used [38,39]. Since this method can only be applied to unoccupied spaces using a tracer gas (e.g., CO_2_), it was convenient to use it in an off-campus period and, therefore, the educational buildings were unoccupied. This decay method is used as a tracer-gas technique to carry out ventilation measurements, and, in this technique, the CO_2_ concentration is increased, emitting this gas into the room and mixing it with the air in the room. Once a sufficient concentration is reached and it is uniform, the decrease of CO_2_ concentration begins, and it is recorded during a given period [40]. The HOBO^®^ MX1102 loggers were the sensors used to measure the CO_2_ concentrations in the field tests. Five CO_2_ concentration measurement sensors were distributed throughout the room during the tests (see Appendix A, Figure A1). These sensors are characterised by a measurement range from 0 to 5000 ppm (accuracy ± 50 ppm ±5% of reading at 25 °C, less than 90% RH non-condensing and 1013 mbar). The sensing method is non-dispersive infrared absorption. 

The data obtained from the field measurements were used to estimate the ACH using Equation (1):

(1) $$ACH=\frac{-1*\;\ln\left(\frac{C_{end}-C_{outdoor}}{C_{start}-C_{outdoor}}\right)}{t_{end}-t_{start}}$$

where *C_end_* is the measured CO_2_ concentrations at the end of the decay curve, *t_end_* is the end time of the decay curve, *C_start_* is the measured CO_2_ concentrations at the start of the decay curve, *t_start_* is the end time of the decay curve, and *C_outdoor_* is the measured CO_2_ concentrations outside of building. The results allow for a comparison of the ACH provided by the different configurations.

In addition, since a VR of 6 ACH (corresponding to ~12.5 L/s per person) is the value recommended by the re-opening guidelines [33] to achieve good IAQ, the results obtained after applying this method were used to select the door and window opening configuration that provides the required ACH value. The location of the sensors (sensor 1–sensor 5) in the classroom during the data collection process is shown in Figure A1, and they were selected to characterise the rooms appropriately.

### 2.3. Monitoring Indoor Environmental Factors under the Selected Ventilation Strategy 

Once the process of characterising the different natural ventilation configurations was completed and the windows and door opening configuration that provides the required VR was selected, indoor environmental conditions were monitored during face-to-face teaching activities with the selected natural ventilation configuration.

Data collection was carried out on two different time periods representative of the room use in quite different conditions: one day in the summer season and the other in the winter season. The duration of the monitoring was adjusted to the teaching activity, which was an exam lasting at least 100 min in both cases. The measurement day, period, duration, and classroom occupancy are shown in Table 1.

In the winter period, the measurement was carried out with 19 students and 2 teachers inside the classroom. In the case of the summer measurement, there were 15 students and 2 teachers. The age range of the student group was between 19 and 27 years old in both cases. All the participants wore clinical or surgical mask during the period of data acquisition. In addition, in order to comply with the COVID-19 Action Plan, class occupancy was limited to 50% and students were seated in their pre-assigned positions to take the exam, keeping the recommended security distance of 1.5 m from each other. So, the students were distributed in the room in such a way as to maximise the distance between them, ensuring a minimum distance of 1.5 m. Moreover, the protocols defined in the plan include that the windows must be open before and after the teaching activity. This IAQ management measure aims to dilute any pollutants present in the air before and after the activity through indoor air renewal.

During the field measurements, data on CO_2_ concentration, air velocity, temperature, and relative humidity were collected. Three of the five HOBO^®^ MX1102 loggers were used for this purpose (Sensors 1, 2, and 3). In addition to the CO_2_ sensors, this instrument contains a temperature sensor, which ranges from 0 to 50 °C, with an accuracy of ±0.21 °C from 0 to 50 °C. The humidity sensor measurements range from 1% to 90% RH (non-condensing) with an error ±2% from 20% to 80% typical to a maximum of ±4.5%, including hysteresis at 25 °C, below 20%, and above 80 ± 6% typical. A hotwire air speed transmitter was used for measuring the indoor air speed with a measuring range from 0.1 to 5 m/s (HD403TS2, Delta OHM, Italy).

**Figure 3:**
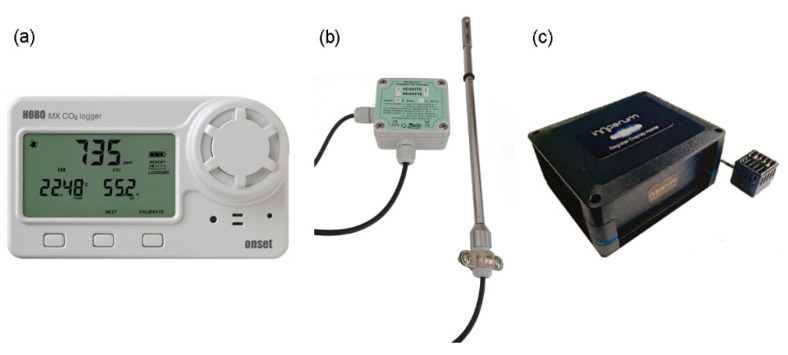
(**a**) HOBO^®^ MX1102; (**b**) HD403TS2, Delta OHM; (**c**) Imperum-R recorder.

The sound pressure level of background noise was measured using an Imperum-R recorder and omnidirectional microphone provided by TECNITAX^®^ Ingeniería (range: 35–115 dBA and frequency from 31.5 Hz to 12.5 kHz). All the IEQ parameters were collected at one-minute sampling intervals during the field monitoring. Figure 3 shows the sensors used in the field monitoring. The locations of the five HOBO^®^ MX1102 loggers, air speed sensor, and acoustic sensor in the classroom during the data collection process are shown in Appendix A (Figure A1).

## 3. Results and Discussion

### 3.1. Natural VR with Different Windows and Door Opening Configuration

**Figure 4:**
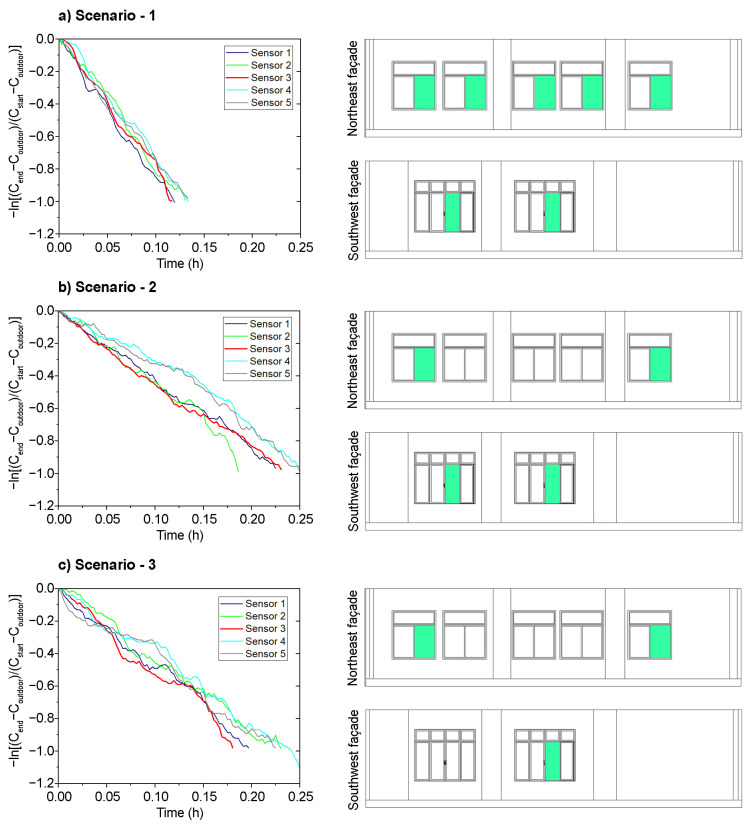
VR according to the ventilation scenario. Open windows are indicated by green shading in the figure.

The results obtained in the three cross-natural ventilation scenarios are shown in Figure 4 and Table 2. The tests were carried out on the same date (in January), and the VRs were quantified with the same outdoor environmental conditions (the outdoor air temperature, RH, and wind speed were 18.5 °C, 47%, and 1.85 Km/h SO-NO, respectively). The tests were carried out during this period following the IAQ recommendations and protocols set out in the Contingency and Action Plan for COVID-19, with the aim of assessing which of the window and door opening configurations provide the required VR.

The duration of the tests depended on the time required for the decrease of the CO_2_ concentration in each tested configuration. This time interval can be seen in Figure 4; the configuration with the shortest decay time provides the highest VR value. In addition, Figure 4 shows the differences between the data recorded in each position by the CO_2_ dataloggers (Figure A1 in Appendix A shows the location of the sensors). The differences observed for those data obtained from each sensor for all the tested configuration scenarios can be explained by their different relative position on the windows and doors for each sensor and the indoor air currents.

Then, from the CO_2_ concentration data recorded on time and after applying the decay method, the VR was calculated for each one of the sensors in all the configurations. The mean VR ((ACH) ®) value was then estimated from the average of the RVs obtained from the sensors in each tested configuration scenario. The average VRs obtained in Ventilation Scenarios 1, 2, and 3 are 7.9, 5.7, and 4.7 ACH, respectively. The windows and door opening scenario that provides the highest ACH is Ventilation Scenario 1. In fact, this scenario is the only one that provides an ACH value that meets the recommendations set out in the guidelines (i.e., 6 ACH). In the other windows and door opening configurations (Ventilation Scenarios 2 and 3), a sufficient ACH value is not achieved at all measurement points in the classroom. Consequently, the window and door opening configuration of Ventilation Scenario 1 was selected for monitoring the indoor environmental conditions in the next phase.

It should be taken into account that this study follows the IAQ management protocols implemented as a result of the Contingency and Action Plan for COVID-19 [34]. Consequently, the conditioning factors of this study include the effect of local indoor and outdoor environmental conditions, which are affected by the IAQ protocols. In this regard, since classrooms have to be naturally ventilated before and after the teaching activities (at least for 1 h), the air temperature and relative humidity levels inside the classrooms are similar to those levels outside them. Therefore, the effect and influence of these factors should be considered if different environmental conditions were applicable on a case-by-case basis for each classroom.

### 3.2. Indoor Environmental Conditions

**Figure 5:**
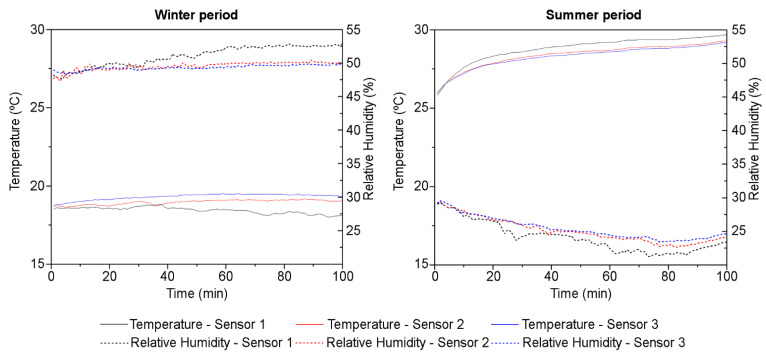
Temperature and humidity data obtained in both winter and summer assessment periods.

This section shows the data obtained from monitoring the teaching activities with the selected natural ventilation strategy. The results are shown below, differentiating be-tween the data collected in the winter and summer seasons. With regard to the temperature and relative humidity measurements, Figure 5 shows the data obtained on both days. The average temperature, RH, and air velocity during the measurement period were 18.9 °C, 50%, and 0.08 m/s in winter, and 28.1 °C, 25.1%, and 0.13 m/s in summer, respectively. These values are representative of the average values in these seasons during the teaching period.

It should be noted that the ventilation protocol of opening the windows before and after teaching activities is critical since it dilutes the pollutant concentration, thus lowering any subsequent dose inhaled by the occupants. However, this procedure also results in indoor temperatures and humidity levels similar to the outdoor environment. The outdoor temperatures during the field measurement periods were 16 °C and 55% HR in winter and 30 °C and 22% HR in summer. Therefore, it can be seen that the outdoor temperature and HR values are not really far from the values measured indoors.

Comparing the temperature values obtained with those recommended by the SINPHONIE guidelines [41], which state that a physically comfortable operating temperature of ~20–26 °C should be maintained in classrooms throughout the year (depending on the season and the outside air temperature), it can be observed that the values obtained are far from the recommended range in both cases. The RH values obtained also fall outside of the reference comfort recommended (42–50% HR) [41]. In both the winter and summer periods, increasing or decreasing the classroom temperature to achieve thermal comfort would require additional energy inputs into heating and/or air conditioning systems.

If these results are compared with other previous related research, it can be observed that these conclusions are in good agreement with those ones. Alonso et al. [42] evaluated the COVID-19 protocol effects on thermal comfort in primary schools in winter in a Mediterranean climate. In their study, Alonso et al. (2021) concluded that the total percentage of discomfort weekly hours will exceed the 80% value, evaluated using various models in naturally ventilated classrooms. This value is above that reported in the year prior to the COVID-19 disease, where the total percentage of discomfort weekly hours was between 50 and 60%.

**Figure 6:**
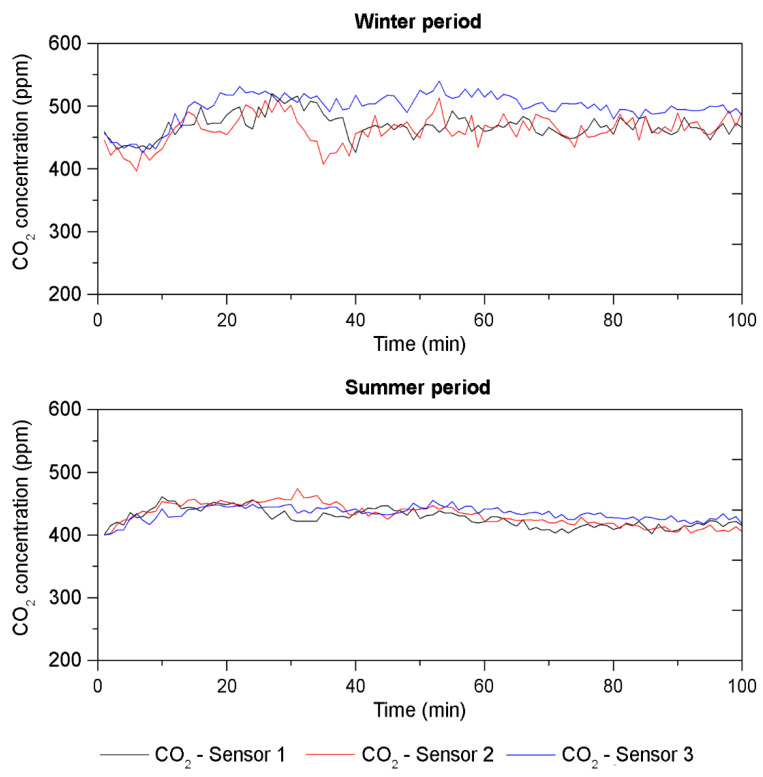
CO_2_ concentration data obtained in both assessment periods.

Regarding the obtained CO_2_ concentration values, Figure 6 shows the data collected in both field measurements. It is worth noting that the previously selected natural ventilation strategy (i.e., ventilation Scenario 3 that provides a VR of 6 ACH) resulted in concentration levels of CO_2_ below 600 ppm throughout the teaching activity. The average concentration values were 476 and 430 ppm in the summer and winter field measurements, respectively. Although the average CO_2_ concentration level obtained was higher in the winter measurement than in the summer data set, it is mainly due to fact that the number of occupants in the classroom during the winter field measurement was higher than in summer.

It should be noted that, if both average CO_2_ concentration levels are compared with the World Health Organization recommended limit (1000 ppm) [43] and REVHA limit (800 ppm) [44], the measured concentration levels were both below the limits.

As expected, occupant density and the type of activity affect the concentration of CO_2_. In this case, the maximum capacity of the classroom was 48 occupants after applying the protocols stated in the COVID-19 Action Plan. Since the occupation during the two field measurements was lower than this value (there was 21 occupants in winter and 17 occupants in summer), the CO_2_ concentration values measured were much lower than the limits. Comparing the occupation ratio per student minimum recommended (i.e., 3.6 m^2^/student), in both scenarios, it was higher: the occupation rate was 9.2 and 11.7 m^2^/student, respectively.

In this context, recent studies on the analysis of natural ventilation strategies show good agreement with those results obtained in this research. Villanueva et al. [45] assessed CO_2_ concentrations in reopening schools and high schools after the lockdown and concluded that the ventilation strategies adopted because of COVID-19 led to substantially improved CO_2_ concentrations compared to previous reports. Specifically, concentrations above 700 ppm were only found in 26% of the classrooms.

Similar conclusions were drawn in the research conducted in kindergartens by Lovec et al. [46]. In their study, their results showed a 30% improvement in the daily average CO_2_ concentration compared to the values measured before the COVID-19 disease. In addition, the research conducted by Meiss et al. [47] on natural ventilation strategies in classrooms concluded that continuous natural cross-ventilation ensured the lowest CO_2_ levels compared to the other scenarios studied. Therefore, cross-ventilation with opening windows and doors is recommended for health emergency situations.

**Figure 7:**
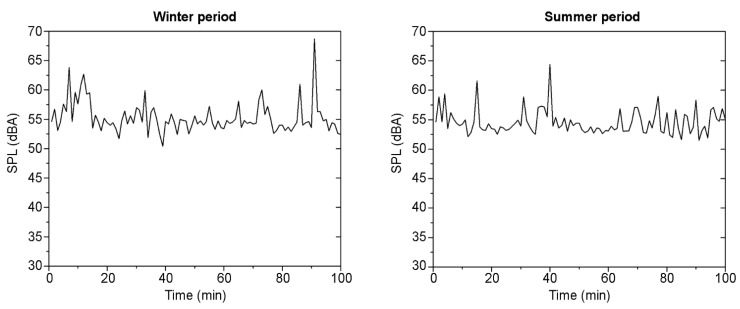
Background noise level (dBA) data obtained in both assessment periods.

With respect to the acoustic environment, the background noise levels measured in the winter and summer seasons are shown in Figure 7. In both cases, the continuous equivalent sound pressure level (LAeq) value obtained in the winter assessment period (56.5 dBA) and in the summer assessment period (55.4 dBA) exceed the recommended LAeq of 35.0 dBA for educational spaces [48,49]. In this case, the obtained LAeq values are similar in the winter and summer periods; therefore, the period in which the measurements were taken did not significantly influence the obtained results. In the case under study, the location of the classroom did have a great influence on the acoustic environment (since it is close to a main street with a high traffic rate). In this sense, high values of background noise levels may cause disruption and loss of concentration among students, but it is due to the specific location of the room.

Regarding this issue, previous studies have shown that noise pollution is a great environmental problem in big cities and results in external noise problems in educational buildings [50,51,52,53,54]. The protocols defined in the COVID-19 Action Plan ensure good air quality by increasing the required VR through the opening of doors and windows, i.e., natural ventilation. However, this measure also results in the background noise level inside the classroom increasing due to external sources (noise outside the building and sources from other areas of the building). In this sense, noise pollution becomes a major concern given that, in cases such as the one analysed in this study, a high SPL of environmental noise has a significant impact on students’ reading and mathematics ability [55,56,57]. Furthermore, if the classroom activity is a process of assessing student learning, as it was during the measurement period, this physical agent may influence the results obtained by the students. Consequently, given the high impact that noise can have on teaching activity, the guidelines for action and adaptation of spaces must consider the impact of this environmental factor.

It is also noteworthy that this research was performed in a case study in a higher education building located in the city of Granada. Therefore, evaluating the effects of other COVID-19-based natural ventilation protocols for other buildings and scenarios (different climates, building locations, etc.) is beyond the scope of this research, and it would be necessary to perform this study with a larger sample.

## 4. Conclusions

The recent global health crisis has led to an increasing concern regarding IEQ, with a particular focus on educational buildings. Indeed, IAQ is an essential factor in the control of the transmission of SARS-CoV-2 within indoor spaces. In this context, educational building administrators face the challenge of managing the IAQ to ensure that indoor spaces are healthy and safe. Therefore, some public guidelines have been established for the reopening of educational buildings. These guidelines recommend implementing natural ventilation strategies that provide a VR of at least 6 ACH. In this research, the impact of the implementation of the COVID-19 protocols was analysed. The field measurements were taken in an educational building in southwest Europe during two periods of the year (winter and summer). As the building has no mechanical ventilation, the required VR can only be achieved through natural ventilation. The strategy for locating the sensors and data collection is described in this research, and the data analysis was performed in three different ventilation scenarios (Ventilation Scenarios 1, 2, and 3) and two different seasonal weather conditions.

Once the window and door opening configuration (VS 1) was selected as the only one that provides an ACH value that meets the recommendations set out in the guidelines for monitoring indoor environmental conditions, the measurements were performed for the next research phase. From the obtained data and as a summary of the main results, it can be observed that the temperature and RH show very different values in both measurement periods. In the winter assessment period, the temperature ranged between 17.8 and 19.5 °C, and between 25.2 and 29.7 °C in the summer assessment period. In the case of the RH, it ranged from 47.3% to 53.1% and from 21.2% to 29.5% in the winter and summer periods, respectively. Note that these values were close to the outdoor values. In the case of the indoor CO_2_ concentrations, they were below 600 ppm in both the summer and winter assessment periods. Given that the recommendations set out in the COVID-19 Action Plan were followed (a VR of 6 ACH was provided naturally through open doors and windows, and the windows were opened before and after the teaching activity), the values obtained were well below the recommended limits. In addition, in terms of acoustic environments, both assessment periods showed a high level of SPL background noise. The SPL values were well above 35 dBA during the entire assessment period on both dates.

This analysis led us to the four following conclusions:

(a) The ventilation scenarios have to be carefully analysed to ensure that they meet the recommendations set out in the guidelines (i.e., 6 ACH). In our study, only Scenario 1 achieved a sufficient ACH value. Thus, it is strongly recommended to perform measurements to set up the correct ventilation scenario.
(b) The public guidelines established in the context of the transmission of SARS-CoV-2 within indoor spaces have an impact on the indoor environmental conditions. Although the CO_2_ concentration levels remained well below the limit during the entire teaching activities, the results from this work show that the impact of the implementation of the COVID-19 protocols on the indoor environmental conditions is significant in regard to thermal and acoustic comfort.
(c) The natural ventilation strategy adopted during the classroom activities significantly affects the thermal and acoustic comfort in the classroom. In this sense, it is clear that it is necessary to keep in mind that indoor spaces must be kept safe and healthy, but strategies must also be provided to ensure this while minimising the impact on the other IEQ factors. Increasing or decreasing the classroom temperature to achieve thermal comfort with the VR recommended by the Spanish government would require additional energy inputs into heating and air conditioning systems.
(d) Educational buildings need to establish a set of preferred ventilation schemes that ensure an adequate IAQ without reducing other performance levels, such as thermal comfort and acoustic environment. Adapting the strategies not only to the characteristics of the classroom but also to the characteristics of the activity will ensure that spaces are used safely and provide equal opportunities for students to continue their education in an appropriate environment.

Finally, it should be noted that it is a critical issue to ensure a good IEQ in order to avoid the further closure of educational buildings in the face of new pandemics. The study of different action scenarios, as well as redesigning or modifying the configurations and systems of buildings, is necessary in order to incorporate new healthier building strategies in the future. The process of redesigning interior spaces and adapting them to new needs will lead to more resilient and efficient buildings, and it will provide a safe environment for teachers and students. This is a further area of research that appears to be necessary in this new context.

## Figures and Tables

**Table 1:** Field monitoring experimental data obtained in the selected classroom.

Measurement Day	Season	Period	Duration	Classroom Occupancy
01/02/2021	Winter	16:00–17:40	100 min	21 (19 students + 2 teachers)
08/07/2021	Summer	10:00–11:40	100 min	17 (15 students + 2 teachers)

**Table 2:** ACH results obtained in each cross-natural ventilation scenario.

Scenario	Sensor 1	Sensor 2	Sensor 3	Sensor 4	Sensor 5	$\bar{ACH}$	σ
Scenario 1	8.6	7.6	8.2	7.7	7.5	7.9	0.47
Scenario 2	6.3	6.2	5.8	5.0	5.1	5.7	0.61
Scenario 3	5.1	4.4	5.5	4.2	4.4	4.7	0.55

## Data Availability

Data are provided upon request to the corresponding author.

## Appendix A

Figure A1 shows the location of the sensor during the VR analysis and the subsequent field measurement in the winter and summer periods.
